# Host Plant Compatibility Shapes the Proteogenome of *Frankia coriariae*

**DOI:** 10.3389/fmicb.2017.00720

**Published:** 2017-05-02

**Authors:** Amir Ktari, Abdellatif Gueddou, Imen Nouioui, Guylaine Miotello, Indrani Sarkar, Faten Ghodhbane-Gtari, Arnab Sen, Jean Armengaud, Maher Gtari

**Affiliations:** ^1^Laboratoire Microorganismes et Biomolécules Actives, Université de Tunis El Manar (FST) and Université de Carthage (INSAT)Tunis, Tunisia; ^2^CEA, DRF, Joliot, Lab Innovative Technologies for Detection and DiagnosticBagnols-sur-Cèze, France; ^3^Department of Botany, NBU Bioinformatics Facility, University of North BengalSiliguri, India

**Keywords:** root exudates, *Frankia*, Proteogenome, symbiosis, signaling pathways

## Abstract

Molecular signaling networks in the actinorhizal rhizosphere select host-compatible *Frankia* strains, trigger the infection process and eventually the genesis of nitrogen-fixing nodules. The molecular triggers involved remain difficult to ascertain. Root exudates (RE) are highly dynamic substrates that play key roles in establishing the rhizosphere microbiome. RE are known to induce the secretion by rhizobia of Nod factors, polysaccharides, and other proteins in the case of legume symbiosis. Next-generation proteomic approach was here used to decipher the key bacterial signals matching the first-step recognition of host plant stimuli upon treatment of *Frankia coriariae* strain BMG5.1 with RE derived from compatible (*Coriaria myrtifolia*), incompatible (*Alnus glutinosa*), and non-actinorhizal (*Cucumis melo*) host plants. The *Frankia* proteome dynamics were mainly driven by host compatibility. Both metabolism and signal transduction were the dominant activities for BMG5.1 under the different RE conditions tested. A second set of proteins that were solely induced by *C. myrtifolia* RE and were mainly linked to cell wall remodeling, signal transduction and host signal processing activities. These proteins may footprint early steps in receptive recognition of host stimuli before subsequent events of symbiotic recruitment.

## Introduction

The cohabitation of plant roots and soil microbes has been shaped by a long and complex coevolutionary process (Morgan et al., 2005), leading to specialized and durable interactions ranging from cheating to altruism (Mendes et al., 2011). The multiple plant-microbe interactions taking place in the rhizosphere are mediated through a dynamic network, via molecular signals secreted as a response to attack in the case of disease, or as a developmental root structure such as in rhizobial and arbuscular mycorrhizal symbiosis (Oldroyd, 2013). In addition to their nutritional value for almost all rhizosphere microbes, root exudates (RE) contain chemoattractants, repellents and/or defensins that play key roles in priming and sustaining molecular dialogs with beneficial and pathogenic microbes (Bais et al., 2006). *Frankia* are nitrogen-fixing soil actinobacteria that are best known for their symbiotic lifestyle with a wide range of dicotyledonous host plants collectively designated as actinorhizal plants (Gtari et al., 2013). These latter are pioneering species that enrich poorly fertile or new soils with nitrogen and organic materials and thus are important plants and shrubs in forestry, agroforestry, and for soil reclamation (Benson and Silvester, 1993). The molecular signaling network leading to infection/nodulation in the nitrogen-fixing symbiosis has been well documented in the case of legume-rhizobium associations. Lipo-chitooligosaccharide or Nod factors are the key signals secreted in response to host plant flavonoids perceived by the compatible rhizobia (Lerouge et al., 1990). These rhizobial Nod factors are sensed by the host plant via LysM-receptor-like kinases (LysM-RLKs), triggering a signal transduction cascade leading to invasion of root cortical cells and, further, to the genesis of functional nodules (Oldroyd, 2013). This host signal transduction cascade has been shown to be common in rhizobial, arbuscular mycorrhizal and also actinorhizal symbiosis (Oldroyd, 2013; Svistoonoff et al., 2013). From the microbial viewpoint of the symbiosis, the analysis of several *Frankia* genomes has failed to reveal the presence of these common canonical *nod*ABC genes (nodA-acyl transferase, nodB-chitin deacetylase, nodC-chitin synthase) (Tisa et al., 2016), as has also been the case for several photosynthetic (Giraud et al., 2007) and non-photosynthetic (Miché et al., 2010) bradyrhizobia. While exceptions exist with two *Candidatus Frankia* genomes generated from nodule metagenomes and where canonical *nod*ABC genes and sulfotransferase gene *nod*H have been reported (Persson et al., 2015; Nguyen et al., 2016). The absence of reliable methods for the genetic manipulation of *Frankia* is an obstacle to the identification of such equivalent and elusive microbial signaling molecules in *Frankia*-actinorhizal symbiosis (Benson et al., 2011).

Proteins are involved in the organization of most biochemical reactions, ranging from metabolism to signal processing. The majority of such proteins operate within large multimeric complexes, through biological networks (Schmidt et al., 2014). Extracellular and surface-associated proteins reflect the physiological state of an organism under a given condition and also indicate the systematic interaction between the organism and its biotic and abiotic environment (Armengaud et al., 2012). Study of the whole proteome is of the utmost importance in gaining a deeper knowledge of the molecular processes regulating the cellular physiology of an organism (Aebersold and Mann, 2003). It has been shown that besides Nod factors and surface polysaccharides, proteins transported through general or host-targeting secretion systems are determinants in establishing and maintaining functional nitrogen-fixing symbiosis for both rhizobia (Fauvart and Michiels, 2008) and mycorrhizal fungi (Doré et al., 2015; Wagner et al., 2015). Previous functional analyses through transcriptomics and proteomics have been limited to comparisons of gene expression patterns between *Frankia* in root nodules and free living cultures (Mastronunzio et al., 2009; Alloisio et al., 2010; Mastronunzio and Benson, 2010), and culture transitions between nitrogen-fixing and non nitrogen-fixing conditions (Alloisio et al., 2007; Bickhart and Benson, 2011; Udwary et al., 2011).

*Frankia coriariae* strain BMG5.1 was isolated from root nodules of *Coriaria japonica* (Gtari et al., 2015; Nouioui et al., 2017). As a member of cluster 2, the host range of this strain is limited to members of the *Coriariaceae, Datiscaceae, Rosaceae*, and *Ceanothus*. Using a high-throughput shotgun proteomic approach, we report here the abundance of *Frankia coriariae* proteins matching first-step receptive recognition and response to host plant stimuli. Symbiotic proteins were identified upon comparative proteomic analysis for the strain grown in media matching compatible, incompatible, and non-host-plant signaling systems, corresponding, respectively, to *Coriaria myrtifolia, Alnus glutinosa*, and *Cucumis melo* RE.

## Materials and methods

### Production of root exudates

*Coriaria myrtifolia* represents the compatible actinorhizal host of *Frankia coriariae* strain BMG5.1, whereas *A. glutinosa* is an incompatible actinorhizal host for this strain. *Cucumis melo* was used in this study because it is taxonomically the closest representative to *Coriaria* for which the whole genome sequence is available. Seeds were incubated overnight in sterile tap water, surface-sterilized with 30% hydrogen peroxide for 30 min and washed several times with sterilized, distilled water. Seeds were then germinated on watered sterile sand at 28°C with a 16-h light period. Depending on the species, 5–15 days after germination, seedlings were aseptically transplanted into a Magenta GA-7 box containing 100 ml Broughton and Dilworth solution supplied with a nitrogen source, i.e., 5 mM KNO_3_ (BD+N) (Broughton and Dilworth, 1971). After 15 days' growth, BD+N medium was replaced by BD medium without nitrogen source (BD-N) and RE were collected after 3–4 weeks of plant growth, filter sterilized through a 0.22 μm polycarbonate membrane and freshly added to BMG5.1 cultures.

### Bacterial growth conditions and proteome preparation

*Frankia coriariae* strain BMG5.1 was grown and maintained in BD-N supplemented with 2.5 mM pyruvate as a carbon source, at 28°C. Ten-day-old cultures were supplemented with one volume (vol/vol) of filter sterilized RE from each plant species and incubated for an additional 5 days. All experiments were performed to obtain three biological replicates. Total cellular protein was measured using the BCA method (Smith et al., 1985). Cellular respiration was assessed using an INT (2-(p- Iodophenyl)-3(p- nitrophenyl)-5- phenyl tetrazolium chloride) reduction assay (IRA) (Prin et al., 1990).

For proteomic analysis, cells were harvested by centrifugation at 13,000 × g for 10 min, resuspended in 90 μl lithium dodecyl sulfate β-mercaptoethanol protein gel sample buffer (Invitrogen, Carlsbad, CA, USA) and incubated at 99°C for 5 min as indicated previously (Hartmann et al., 2014). Proteins from 10 ml culture supernatants were precipitated with trichloroacetic acid. The resulting pellets were resuspended in 50 μl lithium dodecyl sulfate β-mercaptoethanol protein gel sample buffer (Invitrogen, Carlsbad, CA, USA) and heated at 99°C for 5 min. The samples were briefly centrifuged to remove large aggregates before SDS-polyacrylamide gel electrophoresis analysis on 10% Bis-TrisNuPAGE gels (Invitrogen). Volumes of 20 μl of either cellular proteomes corresponding to 60 μg of total proteins, or exoproteome corresponding to a few μg of secreted proteins, were loaded per well. Sodium dodecyl sulfate-PAGE was carried out in 1 × 3-(N-morpholino) propane sulfonic acid solution (Invitrogen) on an XCellSureLock Mini-cell (Invitrogen) under a constant voltage of 200 V for 5 min. Gels were stained for 10 min with SimplyBlue SafeStain, a ready-to-use Coomassie G-250 stain (Invitrogen), and then destained overnight with milliQ water. Polyacrylamide gel bands (equivalent in volume to 100 μl) comprising the entire cellular proteome or exoproteome—one band per entire proteome—were cut and processed for in-gel proteolysis with Sequencing Grade Trypsin (Roche, Meylan, France) in the presence of 0.01% Protease Max reagent (Promega, Madison, WI, USA), as described previously (Clair et al., 2010). Three independent biological replicates were analyzed per treatment.

### Nano liquid chromatography–MS/MS analysis

Peptide digests were resolved on an Ultimate 3000 LC system (Thermo-Scientific, Villebon-sur-Yvette, France) before MS/MS measurements performed with a Q-Exactive HF tandem mass spectrometer incorporating an ultra-high-field Orbitrap analyzer (Thermo-Scientific). Conditions for operating the LC system were as described previously (Dedieu et al., 2011). The Q-Exactive HF tandem mass spectrometer was operated in data-dependent mode comprising a scan cycle initiated with a full scan of peptide ions in the ultra-high-field Orbitrap analyzer, followed by selection of the precursor, high energy collisional dissociation and MS/MS scans on the 20 most abundant precursor ions (Top20). Full scan mass spectra were acquired with an Automatic Gain Control Target set at 3 × 10E6 ions from m/z 350 to 1,800 at a resolution of 60,000. MS/MS scan was initiated at a resolution of 15,000 when the AGC target reached 10E5 ions with a threshold intensity of 83,000 and potential charge states of 2+ and 3+. A 10 s dynamic exclusion was activated throughout the gradient. MS/MS spectra were processed and interpreted with the MASCOT 2.3.02 search engine (Matrix Science, London, UK), with standard parameters as indicated previously (Hartmann and Armengaud, 2014). This analysis was performed against two complementary databases: (i) a complete list of annotated CDS from the draft genome of BMG5.1 (GenBank/EMBL/DDBJ accession number JWIO00000000.1) comprising 4,175 protein sequences, and (ii) a complete list of annotated CDS from the finished genome of Dg1, the *Frankia* symbiont of *Datisca glomerata* (GenBank/EMBL/DDBJ accession number CP002801.1) comprising 4,099 protein sequences. Peptide matches with a score above their peptidic identity threshold were filtered at *P* < 0.05. A protein was only validated when at least two peptides had been assigned to that protein according to the principle of parsimony. Using a previously described approach (Liu et al., 2004; Zivanovic et al., 2009), protein abundance was evaluated by shotgun analysis using MS/MS spectral counts. Normalized spectral count abundance factors were calculated as described by Paoletti et al. (2006) and presented as percentages of total signal.

### Bioinformatics analysis

To study the proteome and exoproteome of BMG5.1, self-customized Perl, Python, and R programs were adopted. STRING server version 10.0 (Szklarczyk et al., 2014) was utilized to obtain knowledge about the biological interactions between the different sets of proteins. Proteins of BMG 5.1 were further distributed among the functional Clusters of Orthologous Groups (COG) categories, and Kyoto Encyclopedia of Genes and Genomes (KEGG) pathway categories were viewed for biochemical features and potential links to life and environment.

CodonW (Peden, 2005) was used to calculate diverse codon and amino acid usage indices such as GC (total guanine and cytosine content of a genome), GC3 (total content of guanine and cytosine present at the third position of the synonymous codons), Enc (effective number of codons), CAI (codon adaptation index; a direct measurement of the expression level of protein encoded by a particular gene), Fop (frequency of optimal codons), RSCU (relative synonymous codon usage), aromaticity, and hydrophobicity (Sen et al., 2008; Roy et al., 2015). These parameters can help us by providing a plethora of information regarding the codon bias nature of symbiosis-related genes and its implication in the lifestyle of the strain studied under different conditions. To further analyze the amino acid usage pattern of the core genes, aromaticity, gravy score, and protein biosynthetic cost were correlated with each other. The tRNA adaptation index (tAI) (dos Reis et al., 2004) was calculated via tRNAscan-SE and codonR software. The tAI of a gene predicts the usage of those codons that perfectly match with anti-codons of major isoaccepting tRNAs present in the studied organism. To assume the biosynthetic energy cost of each protein of BMG5.1, DAMBE software (Xia and Xie, 2001) was used, which calculates the protein energy cost based on the algorithm proposed by Akashi and Gojobori (2002). Pfam server (http://pfam.xfam.org/) was used to predict the protein domains present in the particular protein sets expressed under different RE treatments.

### Data repository

The mass spectrometry proteomic data were deposited at the ProteomeXchange Consortium (http://proteomecentral.proteomexchange.org) via the PRIDE partner repository (Vizcaíno et al., 2016) with the data set identifiers PXD005979 and DOI 10.6019/PXD005979 (cellular proteome data), and PXD005980 and DOI 10.6019/PXD005980 (exoproteome data).

## Results

### High-throughput bacterial proteomics

A total of 149,629 and 144,213 MS/MS spectra were assigned on the basis of the BMG5.1 and Dg1 databases, respectively. As expected, the highest assignment rate was obtained with the genome of the strain used in this study. However, some additional spectra could be assigned using the Dg1 database because of missing gene sequences or annotations in the first genome. Indeed, the number of unique peptide sequences was 6,627 peptides when the BMG5.1 database was used, while 5,879 peptides were found against the Dg1 database. When merging both data sets, the additional Dg1 database search allowed the detection of 918 additional unique peptide sequences. The whole set of peptides indicated the presence of 1,330 proteins identified with at least two peptide sequences and 572 further protein candidates detected with only one peptide sequence (Supplementary Tables S1, S2). The high-throughput approach used here, which relies on one of the most recent tandem mass spectrometers, allows a deep characterization of bacterial proteins. An average of 21% of each protein sequence was covered by certified tryptic peptides, and a high redundancy in terms of spectral counts was obtained with an average of 166 MS/MS spectra recorded per protein. The abundances of the proteins were assessed by their spectral counts in the different replicates and conditions. Among the proteins specifically detected using the Dg1 database, and thus wrongly annotated in the BMG5.1 database (Supplementary Tables S1, S2), we noted the presence of high abundance proteins: three transcriptional regulators (KLL11059.1; WP_013874517.1; KLL10836.1) and several hypothetical proteins (WP_043603927.1; WP_050803503.1; WP_050803326.1; WP_050803366.1; WP_013875127.1). Remarkably, the DNA-directed RNA polymerase subunit beta (WP_013875121.1) and ATP-dependent Clp protease ATP-binding protein (WP_013875507.1) sequences were well covered with our proteogenomic approach based on the use of the Dg1 closely related genome information, with 60 and 36 different peptides, respectively. Table 1 summarizes the proteomic data predicted from genome sequences and experimentally determined in this study.

**Table 1:** **Protein abundance, as predicted from Dg1 and BMG5.1 genome sequences and detected experimentally for ***Frankia coriariae*** strain BMG5.1**.

	**Cell proteome**		**Exoproteome**	
	**BMG5.1 database**	**Dg1 database**	**BMG5.1 database**	**Dg1 database**
Genome-based prediction	1,320	1,435	79	84
Experimentally detected	1,626	1,346	78	83

### Next-generation proteomics of the BMG5.1 strain grown in BD-N medium

**Figure 1:**
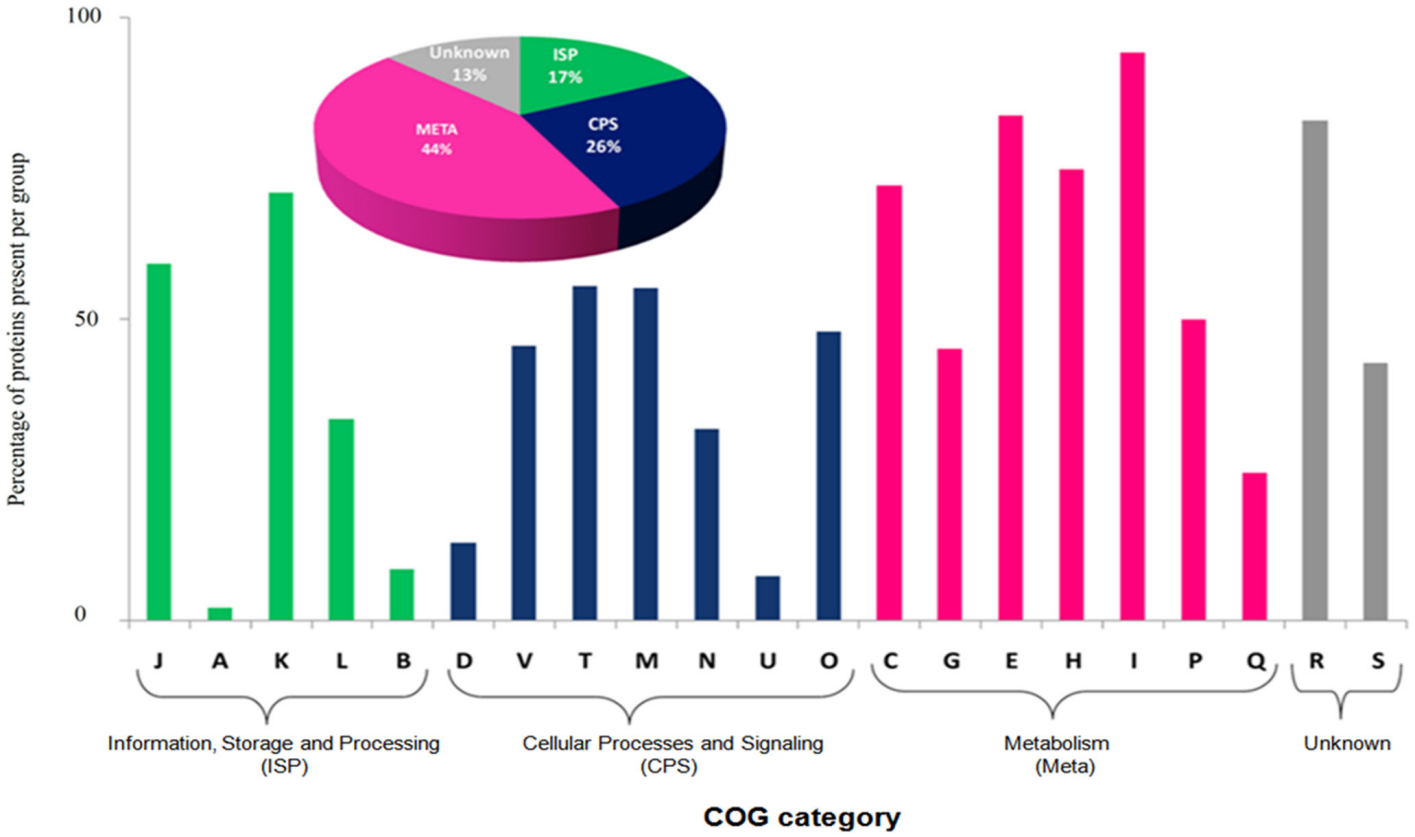
**Functional classification based on Clusters of Orthologous Groups (COG) of BMG5.1 proteome**. Proteins were distributed among the COG categories on the basis of percentage protein abundance. J, Translation, ribosomal structure and biogenesis; A, RNA processing and modification; K, Transcription; L, Replication; recombination and repair; B, Chromatin structure and dynamics; D, Cell cycle control, cell division, chromosome partitioning; V, Defence mechanisms; T, Signal transduction mechanisms; M, Cell wall/membrane biogenesis; N, Cell motility; U, Intracellular trafficking, secretion, and vesicular transport; O, Posttranslational modification, protein turnover, chaperones; C, Energy production and conversion; G, Carbohydrate transport and metabolism; E, Amino acid transport and metabolism; F, Nucleotide transport and metabolism; H, Coenzyme transport and metabolism; I, Lipid transport and metabolism; P, Inorganic ion transport and metabolism; Q, Secondary metabolites biosynthesis, transport and catabolism, R, General function prediction only; S, Function unknown.

**Figure 2:**
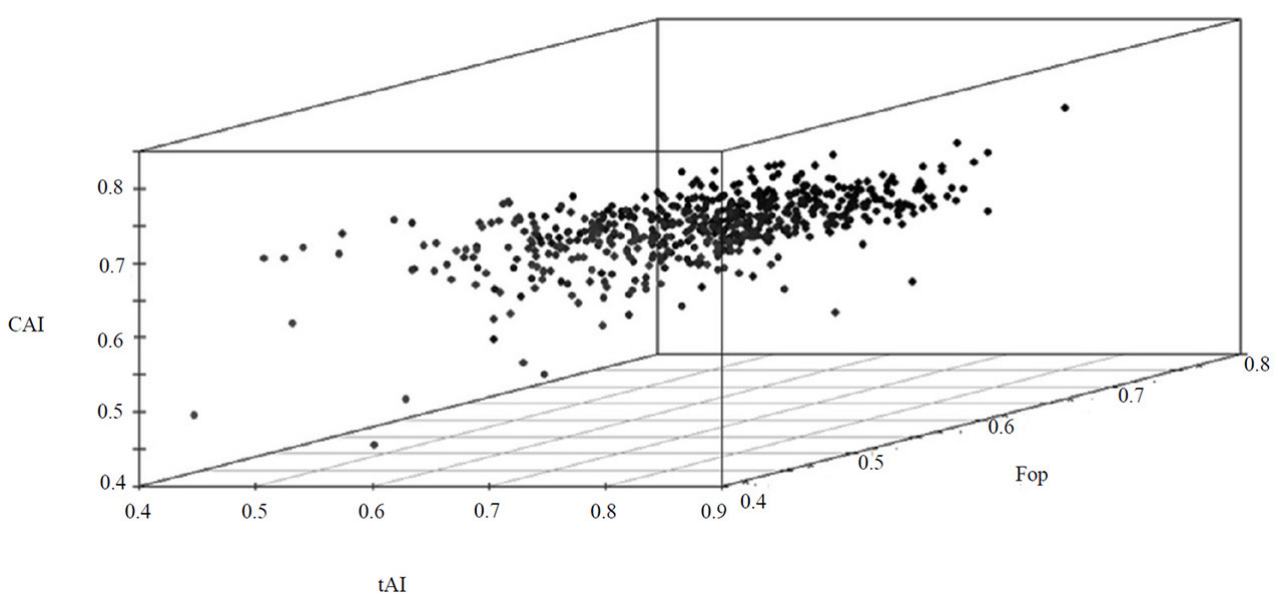
**A 3D plot of CAI, Fop and tAI values for the BMG5.1 proteome, revealing the positive correlation between the three parameters**.

The most abundant proteins of the BMG5.1 cellular proteome comprise housekeeping proteins involved in carbohydrate metabolism, nitrogen fixation and assimilation, and DNA replication, repair, transcription and translation (Figure 1). Proteins encoded by genes with unassigned functions were also recovered using our high-throughput proteomic analysis. An overall codon usage and amino acid usage study of these MS/MS-detected proteins revealed a high positive correlation (*p* < 0.001) between GC and CAI whereas a statistically significant negative correlation was obtained between GC and Enc (*p* < 0.001). On further analysis, we found a positive correlation between CAI and aromaticity as well as aromaticity and energy cost. A similar positive correlation was obtained between CAI, Fop, and tAI (Figure 2).

**Figure 3:**
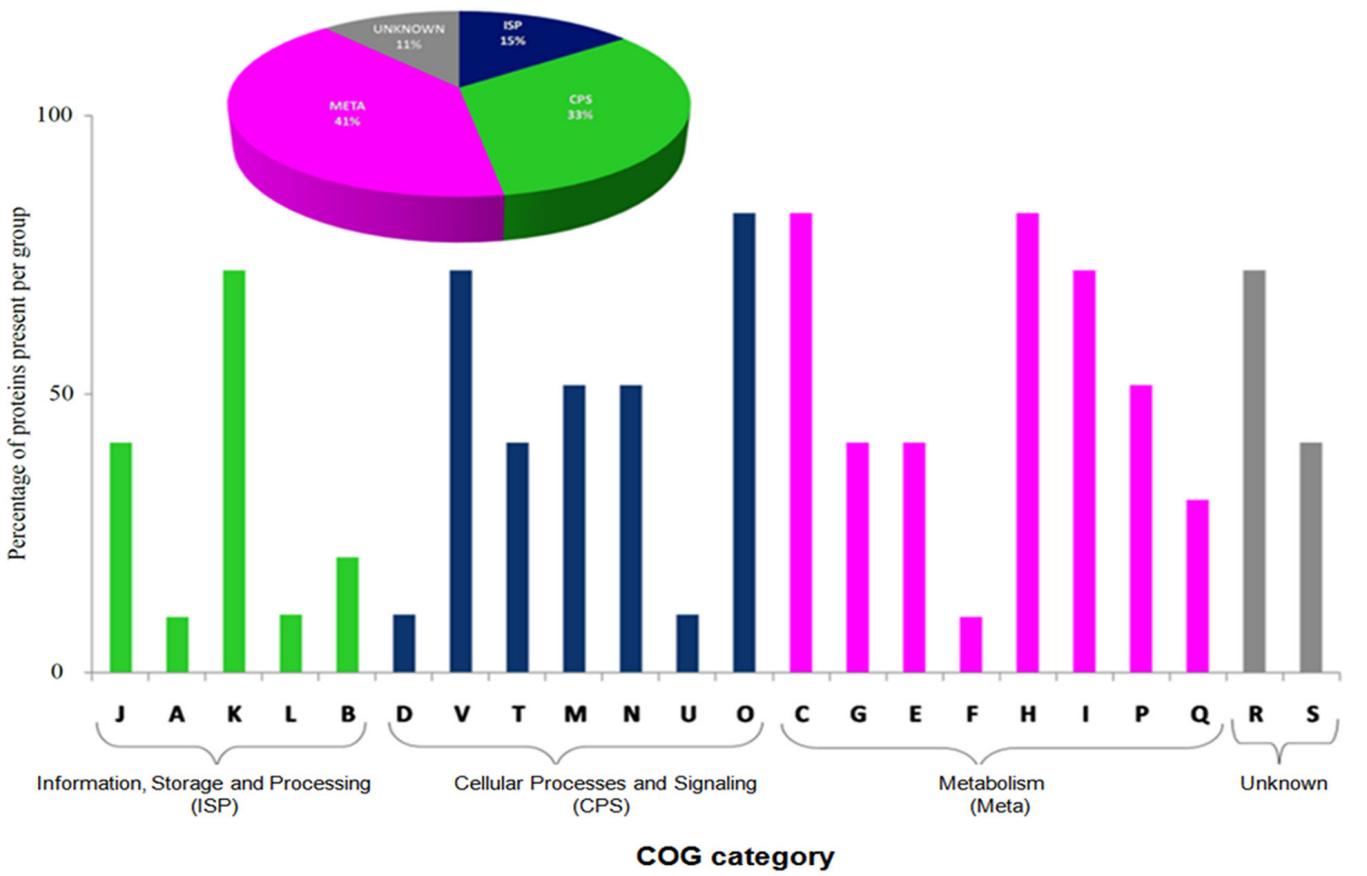
**Functional Clusters of Orthologous Groups (COG) classification of the BMG5.1 exoproteome, showing “metabolism” as the major category**. The plot was generated on the basis of percentage of protein abundance in the different COG categories. J, Translation, ribosomal structure, and biogenesis; A, RNA processing and modification; K, Transcription; L, Replication, recombination, and repair; B, Chromatin structure and dynamics; D, Cell cycle control, cell division, chromosome partitioning; V, Defence mechanisms; T, Signal transduction mechanisms; M, Cell wall/membrane biogenesis; N, Cell motility; U, Intracellular trafficking, secretion, and vesicular transport; O, Posttranslational modification, protein turnover, chaperones; C, Energy production and conversion; G, Carbohydrate transport and metabolism; E, Amino acid transport and metabolism; F, Nucleotide transport and metabolism; H, Coenzyme transport and metabolism; I, Lipid transport and metabolism; P, Inorganic ion transport and metabolism; Q, Secondary metabolites biosynthesis, transport, and catabolism; R, General function prediction only; S, Function unknown.

The BMG5.1 exoproteome (Supplementary Tables S3, S4) contains a total of 98 proteins. Of these, 70 are shared with the cellular proteome, although more abundant in the exoproteome, while 28 are unique to the secreted proteome, mainly involved in essential physiological activities such as transcriptional regulation, ribosome formation, RNA degradation, oxidative phosphorylation, glycolysis/gluconeogenesis, and transport systems (Supplementary Table S5). Nitrogen fixation-related proteins were not detected in the exoproteome fraction. Thus, the exoproteome contains proteins that are in particular required for regulating the most essential biochemical pathways related to the existence of the organism itself. Cello server (Yu et al., 2006) queried with this list of exoproteins predicted preferentially cytoplasmic (74%), membrane-bound (13%), and extracellular (13%) proteins. It is worth noting that an ATP-dependent protease and a proteasome have been detected systematically in BMG5.1 culture supernatants. The COG category analysis detected “metabolism” as major functional category (Figure 3). The KEGG pathways are, broadly, carbohydrate metabolism, ribosome formation, amino acid metabolism, and signal transduction.

A detailed codon usage analysis of exoproteome-encoding genes revealed that they preferentially favor the use of GC-enriched codons. Negative correlation between ENc and GC3 verified GC compositional constraint as a major factor driving codon usage. However, significant positive correlation between tAI, Fop, and CAI (*p* < 0.001) indicates the favored utilization as well as the translational adaptability of major optimal codons. The energy cost of these proteins showed a significant positive correlation with aromaticity and tAI (*p* < 0.001). This result indicates the favored usage of costly aromatic amino acids in these proteins. Thus, the exoproteome of BMG5.1 is composed of costly aromatic amino acids, highlighting the high relevance of these amino acids in symbiotic association and for nitrogen fixation. Proteins that were detected in the exoproteome but not in the cellular proteome (*n* = 28) were further analyzed for aromatic amino acid counts, which were found to be lower than for the proteins that were shared with cell proteins.

### Next-generation proteomics of the BMG5.1 strain grown in the presence of root exudates

**Figure 4:**
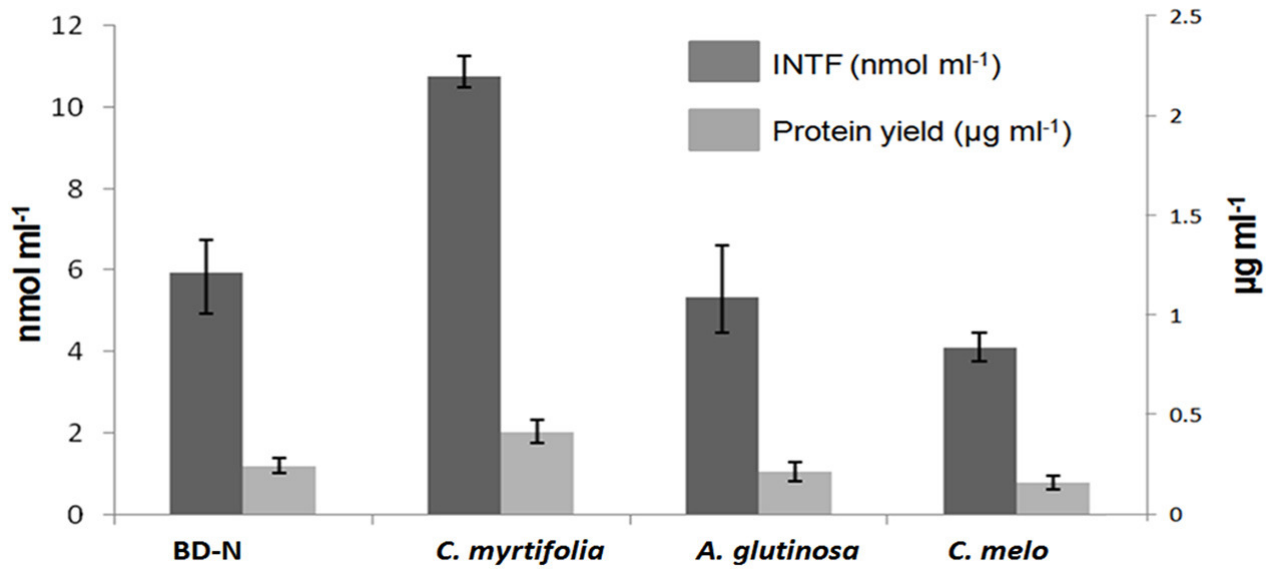
**The effect of ***C. myrtifolia, A. glutinosa***, and ***C. melo*** root exudates on ***Frankia coriariae*** BMG5.1 growth yield, as determined by protein yield and respiration assay using a spectrophotometric measurement of the reduction of 2-(p-iodophenyl)3-(p-nitrophenyl)-5-phenyl-tetrazolium chloride (INT) to iodo-nitrophenyl formazan (INTF)**. Strain BMG5.1 was grown in presence of filter sterilized root exudates from the indicated plant species. BD-N (nitrogen-free Broughton and Dilworth medium) was used as control.

Strain BMG5.1 grows better in the presence of *C. myrtifolia* RE than in BD-N medium, followed by *A. glutinosa* then *C. melo* RE, as shown by total cellular protein yield and cell respiration (IRA) assays, the two reliable indicators of biomass production and metabolic activities (Figure 4).

To check potential interference of plant proteins derived from RE, we first interpreted MS/MS spectra for the identification of peptides derived from *C. melo* RE in comparison with the exoproteome of BMG5.1 grown in *C. melo* RE. The proteins obtained by acid precipitation from 40 ml RE were quantitatively insignificant, as verified by SDS-PAGE and by the very low level of MS/MS spectra recorded by our shotgun procedure (*n* = 373), which corresponded to 77 peptide sequences. The average MASCOT score of proteins from *C. melo* RE is 28.9, a very low value compared to the score for strain BMG5.1 grown in *C. melo* RE, 45.6, thus indicating a rather low confidence for exudates spectrum assignment. Further interpretation of BMG5.1 culture supernatants produced in the presence of *C. melo* RE against *Frankia* (Supplementary Table S1) and *C. melo* databases indicated that MS/MS spectra allocations wholly derived from *Frankia* peptides.

Comparison of BMG5.1 proteomes in BD-N medium with those obtained in the presence of all three RE revealed a total of 404 common proteins in the cellular proteome of BMG5.1 (while the number was 376 in Dg1 with the same conditions). When the RE were used individually, these counts were reduced to 371 (BMG5.1) and 343 (Dg1) in *A. glutinosa* RE, whereas and 308 (BMG5.1) and 292 (Dg1) in *C. melo* RE. However, as expected, the proteome size was higher (470 for BMG5.1; 438 for Dg1) using RE of *C. myrtifolia*, which is the natural host of the BMG5.1 strain.

These differentially expressed protein sets did not reveal a significant variation with respect to protein domains. Among the 404 proteins of BMG5.1 expressed under the influence of cumulative REs (which henceforth will be called as cREEP), we found a total of 37 functional domains. Of these 37 domains, there were four that were the most prevalent within the majority of the cREEP.

These four domains were PAS (pfam00989, pfam13426, pfam08447, pfam08448), GGDEF (pfam00990), HisKA (pfam00512, pfam07568, pfam07730), and cytochrome p450 (pfam00067), associated with bacterial signal transduction. Other domains were found to be involved in metabolism, especially carbohydrate metabolism. Some examples of these domains, present at moderate levels among the cREEP (supplementary Table S6), are Gp_dh_N (glyceraldehyde 3-phosphate dehydrogenase, NAD binding domain) (pfam00044), Gln-synt_C (glutamine synthetase, catalytic domain) (pfam00120), PGI (phosphoglucose isomerase) (pfam00342), 6PGD (6-phosphogluconate dehydrogenase, C-terminal domain) (pfam 00393), PGM_PMM_IV (phosphoglucomutase/phosphomannomutase, C-terminal domain) (pfam00408), and UDPG_MGDP_dh (UDP-glucose/GDP-mannose dehydrogenase family, central domain) (pfam00984).

**Figure 5:**
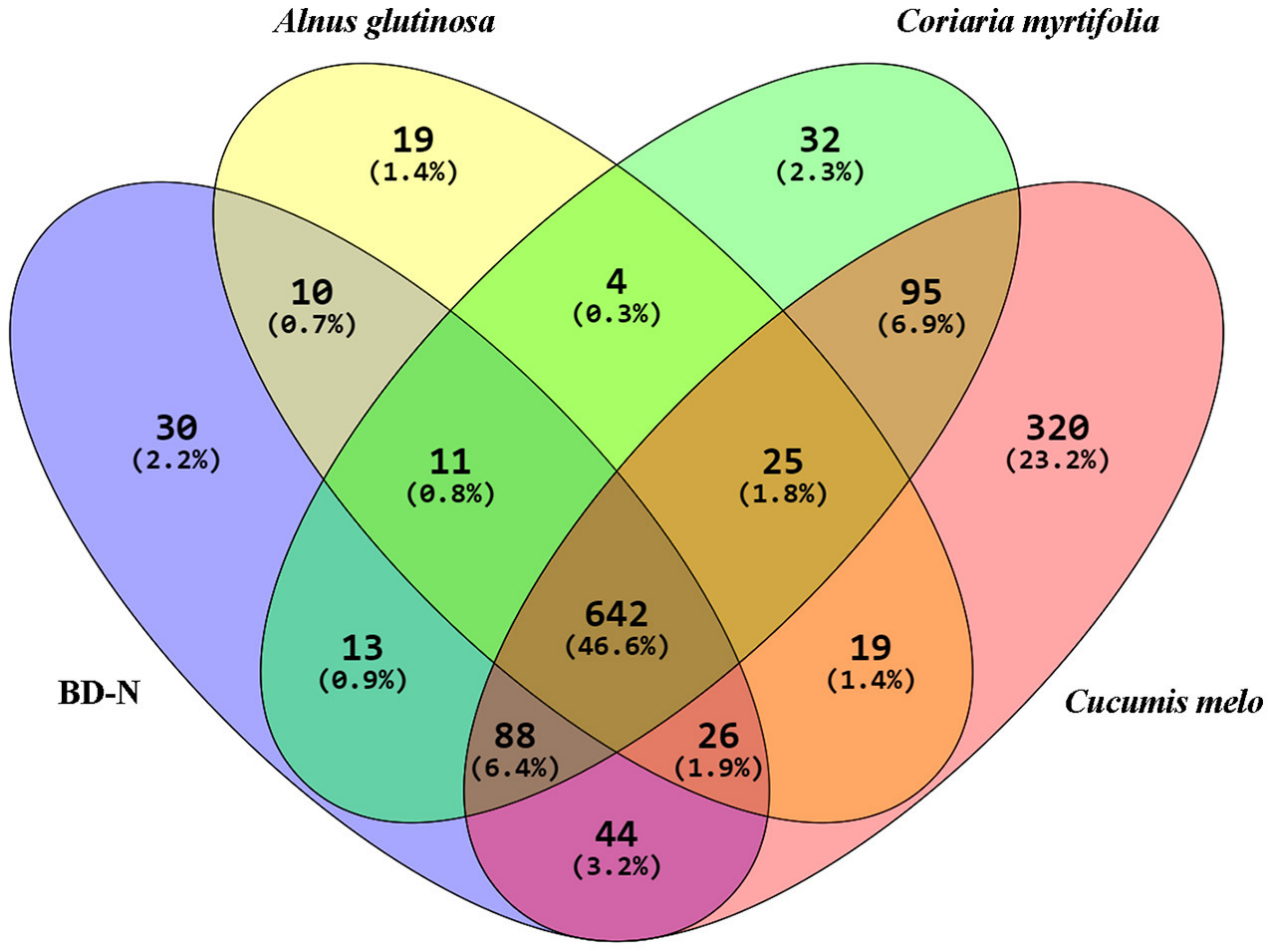
**Venn Diagram showing the distribution of BMG5.1 proteins grown in BD-N (nitrogen-free Broughton and Dilworth medium) and in the presence of root exudates from the indicated plant species**. A total of 642 proteins were detected under all conditions. These proteins were found to be mostly housekeeping proteins involved in metabolism and signal transduction.

The clustering coefficients and *P*-values obtained from biological interaction study of these cREEPs clearly indicate that they are biologically connected and the clustering coefficients were statistically supported. The functional COG category analysis of these proteins showed “metabolism” and “cellular process & signaling” to be the major functional groups. The overall unique and shared proteins of the cellular proteome and exoproteome expressed under different root exudates and BD-N are illustrated in a Venn diagram in Figure 5.

**Figure 6:**
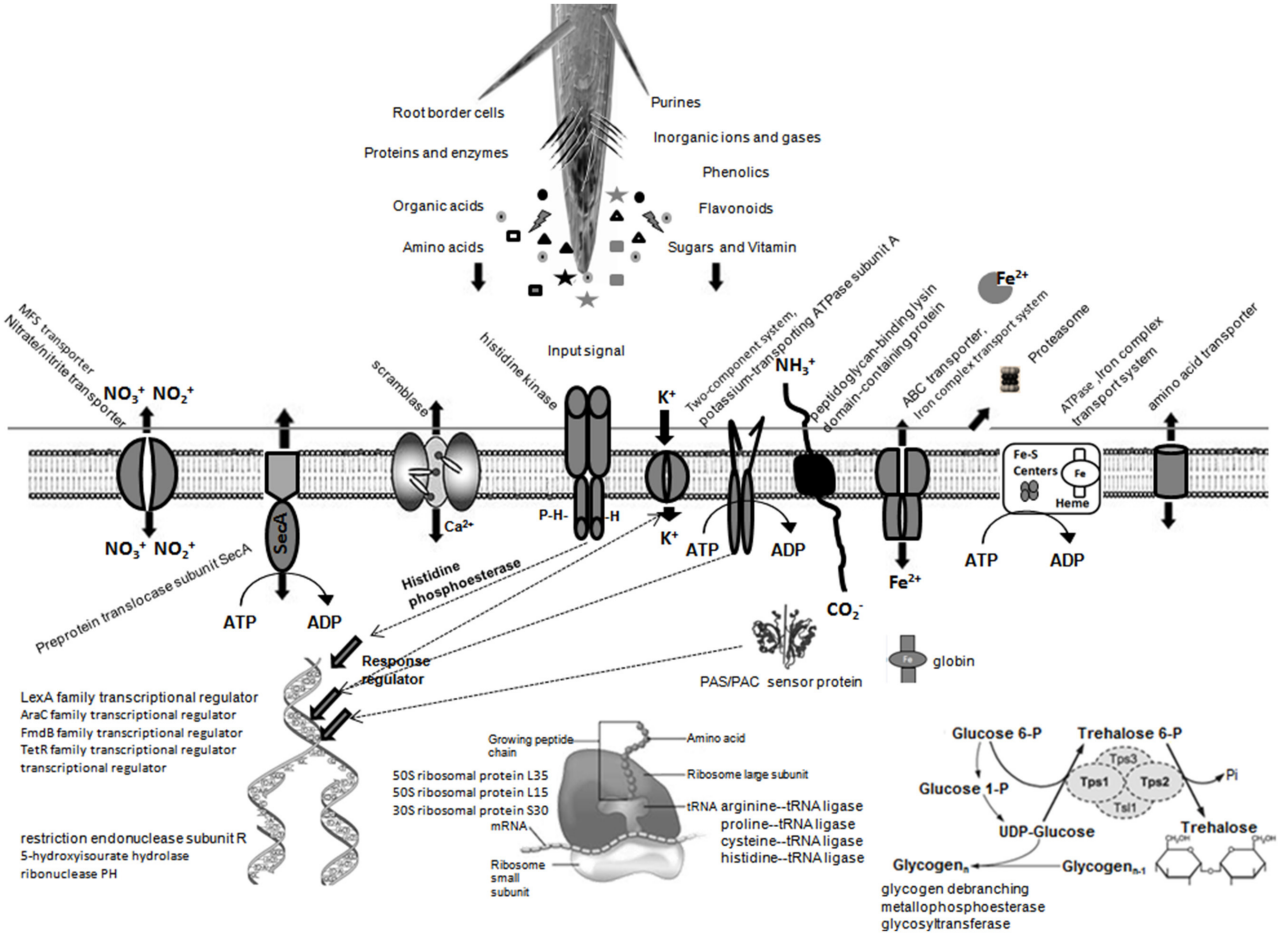
**A predictive overview of the main alterations to strain BMG5.1 cellular processing in the presence of ***C. myrtifolia*** root exudates as expected from COG and KEGG functional assignments of the induced proteins**.

Compared with the BMG5.1 proteome in BD-N medium, 83 unique proteins were induced for the BMG5.1 proteome in the presence of *C. myrtifolia* RE (Table 2). Among the above-mentioned proteins, 46 and 24 were shared with the BMG5.1 proteome in the presence of *A. glutinosa* and *C. melo* RE, respectively, while 32 were unique to *C. myrtifolia* RE. Cello server predicted the cellular localization of these 32 *C. myrtifolia* RE unique proteins to be cytoplasmic (55%), membrane bound (25%), and extracellular (20%). No signal peptide was detected among these proteins. Positive correlation among CAI, tAI and Fop of these protein-coding-genes also implies maximum usage of translationally optimal codons. Figure 6 illustrates the main alterations of BMG5.1 cellular processing when grown in *C. myrtifolia* RE.

**Table 2:** **Proteins induced in ***Frankia*** strain BMG5.1 grown in root exudates**.

**Descriptions with Accessions (Dg1/BMG5.1)**	**Associated pathway (KEGG, BRITE)**	***C. myrtifolia***	***A. glutinosa***	***C. melo***
LexA family transcriptional regulator (KLL12894.1)	DNA repair and recombination	+	+	+
AraC family transcriptional regulator (KLL13116.1)	Base excision repair; transcriptionalregulator	+	+	−
restriction endonuclease subunit R, partial (KLL09521.1)	Hydrolaseactivity	+	+	+
5-hydroxyisourate hydrolase (KLL11137.1)	Nucleotidemetabolism	+	−	−
ribonuclease PH (WP_013872713.1)	Nucleotidyltransferaseactivity	+	−	−
FmdB family transcriptional regulator (KLL11411.1)	Transcriptionalregulator	+	−	−
TetR family transcriptional regulator (KLL12053.1)	Transcriptionalregulator	+	+	+
transcriptionalregulator (WP_052914259.1)	Transcriptionalregulator	+	+	+
50S ribosomal protein L35 (WP_013874486.1/KLL10220.1)	Ribosome formation- Genetic Information Processing; Translation	+	+	−
30S ribosomal protein S30 (KLL12035.1)	Ribosome formation- Genetic Information Processing; Translation	+	+	+
50S ribosomal protein L15 (WP_047221577.1)	Ribosome formation- Genetic Information Processing; Translation	+	+	+
arginine–tRNA ligase (WP_043602701.1)	Aminoacyl-tRNAbiosynthesis	+	+	−
proline–tRNA ligase (WP_013875411.1)	−	+	−	−
cysteine–tRNA ligase (WP_013875400.1)	−	+	+	−
histidine–tRNA ligase (WP_013875269.1)	−	+	+	−
amidohydrolase (WP_043606140.1)	Acting on carbon-nitrogen bonds, other than peptide bonds	+	+	−
prephenatedehydrogenase (WP_013873190.1/KLL11797.1)	Phenylalanine, tyrosine and tryptophan biosynthesis	+	−	−
aminoacidtransporter (KLL12013.1)	Putative aminoacidtransporter	+	−	−
GlcNAc-PI de-N-acetylase (KLL11416.1)	GPI biosynthesis	+	−	−
peptidoglycan-binding lysin domain-containing protein (WP_013872588.1)	−	+	−	−
scramblase (WP_013875539.1)	−	+	−	−
acetyl-CoAacetyltransferase (WP_043605993.1)	Enzymaticactivity	+	+	−
aldo/keto reductase (WP_050803659.1/KLL11374.1)	Enzymaticactivity	+	+	−
metallophosphoesterase (WP_013874361.1/KLL10534.1)	Enzymaticactivity	+	−	+
peptidase (KLL10005.1.1)	Proteasome	+	+	+
oxidoreductase (WP_013874345.1/KLL10011.1)	Citrate cycle	+	+	+
cyclopropane-fatty-acyl-phospholipid synthase (KLL12366.1)	Methyltransferases	+	−	−
3-oxoacyl-ACP synthase (KLL12268.1)	Fatty acid biosynthesis and metabolism	+	−	+
nitrilotriacetatemonooxygenase (WP_013873307.1)	FMN-dependentoxidoreductase	+	+	+
glycogendebranching enzyme (WP_043606277.1)	Glycosylases	+	+	−
haloaciddehalogenase (KLL11333.1)	Glyoxylate and dicarboxylatemetabolism;Biosynthesis of secondary metabolites	+	−	−
globin (KLL11541.1)	Heme binding; oxygen binding	+	−	−
membrane protein (KLL11693.1)	Bacterialsecretion system	+	+	+
preproteintranslocasesubunitSecA (KLL10487.1)	Quorum sensing;Proteinexport;Bacterial secretion system	+	+	+
ABC transporter (WP_013872693.1)	Ironcomplex transport system	+	+	−
ATPase (WP_043607340.1)	Ironcomplex transport system	+	+	−
MFS transporter (KLL10396.1)	Nitrate/nitrite transporter	+	+	+
NADH:ubiquinone oxidoreductase subunit H, partial (KLL12793.1)	Nitrotoluenedegradation;Carbonmetabolism	+	+	+
FMN-dependent NADH-azoreductase (KLL11873.1)	Oxidoreductases; Acting on other nitrogenous compounds as donors	+	+	−
SDR familyoxidoreductase (WP_050803541.1)	−	+	−	−
sulfate adenylyltransferase (KLL12588.1)	Purine and sulfurmetabolism	+	+	−
quinolinatesynthetase (WP_013874400.1)	−	+	−	−
sulfurtransferase (WP_013871733.1)	Sulfurrelay system	+	−	−
phosphoribosylformylglycinamidine synthase II (WP_050803648.1)	−	+	−	−
NADPH-dependent FMN reductase (WP_013871832.1)	Riboflavinmetabolism	+	−	−
histidine kinase (WP_013872222.1/KLL10514.1)	Signaling	+	+	−
putative PAS/PAC sensorprotein (WP_013874756.1)	Signaling	+	−	−
glycosyltransferase family 1 (WP_013872309.1/KLL13063.1)	Starch and sucrose metabolism	+	+	−
glycosyltransferase (KLL09812.1)	Starch and sucrose metabolism	+	−	−
potassium-transporting ATPase subunit A (KLL12342.1)	Two-component system	+	−	−
NADPH-dependent F420 reductase (WP_013874441.1)	Uncharacterized	+	−	−
acetoacetyl-CoAsynthetase (WP_050803816.1)	Valine, leucine and isoleucine degradation	+	+	−
pyridoxamine 5'-phosphate oxidase (KLL12235.1)	Vitamin B6 metabolism	+	+	+
carboxymethylenebutenolidase (WP_050803476.1/KLL11047.1)	Biosynthesis of secondary metabolites; Microbial metabolism in diverse environments; Chlorocyclohexane and chlorobenzene degradation	+	+	−
Hypotheticalprotein^a^	−	+	+	+
Hypotheticalprotein^b^	−	+	+	−
Hypotheticalprotein^c^	−	+	−	+
Hypotheticalprotein^d^	−	+	−	−

*The datasets are a compilation of proteins generated on the basis of the Dg1 and BMG5.1 genome databases*.

a *KLL11549.1, KLL10188.1, KLL10250.1, WP_043604429.1, and WP_047221896.1*.

b *WP_013875584.1, KLL10160.1, WP_013874846.1, WP_052914782.1, WP_013872277.1, WP_043607069.1, WP_013873180.1, WP_050803611.1, WP_050803462.1, and WP_050803545.1*.

c *WP_052914777.1, KLL13066.1, and KLL09907.1*.

d *WP_047221199.1, WP_052914905.1, KLL12041.1, KLL11841.1, KLL11815.1, KLL11583.1, KLL11404.1, KLL11347.1, KLL10248.1, WP_013875131.1, and KLL09806.1*.

## Discussion

### General characteristics of the *Frankia coriariae* strain BMG5.1 proteogenome

The main characteristic of the BMG5.1 shotgun proteome obtained in the present study is its adaptation to the costly aromatic amino acids, which may be of relevance for bacteria with a particular lifestyle in a nitrogen-fixing symbiotic association (Prasad et al., 2000; Randhawa and Hassani, 2002).

As previously reported for *Frankia casuarinae* (Mastronunzio et al., 2008, 2009), the BMG5.1 exoproteome was determined to be tiny, with few secreted proteins. The presence of extensive cell lysis activity was attributed as responsible for the existence of several cellular proteins in the secreted fraction (Mastronunzio et al., 2009). The total absence of signal peptide proteins and the detection of an ATP-dependent protease and a proteasome at higher level in BMG5.1 supernatant, further strengthen this hypothesis. It has been shown that some ATP-dependent proteases are essential elements of the complex regulatory networks enabling the viability of cells under adverse environmental conditions (Gottesman, 1996).

COG analysis attributed most of the secreted proteins to metabolism, especially carbohydrate and amino acid metabolism and transport, as a major functional category. This finding fully supports the findings of Udvardi and Poole (2013), who reported that nitrogen-fixing associations require smooth integration between the bacterial and plant “metabolism–transportation system” for fluid exchange of the metabolic products across the plant and bacterial membranes.

### Root exudates alter the *Frankia coriariae* BMG5.1 proteogenome

RE derived from BMG5.1-compatible (*C. myrtifolia*), BMG5.1-incompatible (*A. glutinosa*), and the non-actinorhizal (*C. melo*) host plants differ both qualitatively and quantitatively (Badri and Vivanco, 2009). RE contain high molecular weight components such as mucilage (polysaccharides) and proteins, and low molecular weight components such as amino acids, organic acids, sugars, and other secondary metabolites (Badri and Vivanco, 2009), which, besides their nutritional value, act as chelators of poorly soluble mineral nutrients and as chemoattractant signals to microbes (Haichar et al., 2014). Thus, *Frankia* growth depends on the ability of a given strain to assimilate nutrients, to tolerate toxic compounds, and to further interact with signaling molecules of a given RE (Turner et al., 2013). A study based on a plant-trapping assay demonstrated the occurrence of *Frankia* in the host and non-host rhizosphere (Smolander and Sarsa, 1990), with a rapid fall in its infective units outside its compatible host area (Benson and Silvester, 1993). Here we showed that RE-induced proteins share some functional domains essential for signaling mechanisms. For instance, GGDEF, HisKA, and PAS domains are associated with bacterial two-component signaling systems (Sarkar et al., 2016). These domains comprise the periplasmic sensory domains with important enzymatic functionalities. They remain associated with the diguanylate cyclase/phosphodiesterase activity of bacteria, which catalyze the synthesis and hydrolysis of the cyclic diguanylate (c-di GMP). This further helps regulation of extracellular polysaccharide formation, the cell development program, and other signaling cascade mechanisms (Galperin, 2004). Among the moderately present domains, Gp_dh_N, Gln-synt_C, PGI, 6PGD, PGM_PMM_IV, and UDPG_MGDP_dh together help in bacterial metabolism, especially carbohydrate metabolism (Saier and Reizer, 1992; Postma et al., 1993; Saier et al., 1995), and may act to bind the bacterial cells to those of the host (Chagnot et al., 2013). Thus, both metabolism and signal transduction are the dominant activities for BMG5.1 under the different RE conditions tested.

The RE-induced proteins represent an overview of the deeply affected metabolic pathways footprinting shifts of the *Frankia* strain from aposymbiotic to rhizosphere, and then from rhizosphere to a symbiotic lifestyle. This indicates (i) adaptation to rhizosphere colonization, which was also induced by the *A. glutinosa* and *C. melo* RE, and (ii) early receptive steps as recognition of host stimuli prior to triggering the root infection and nodulation processes, which was obtained only with *C. myrtifolia* RE.

### Induced proteins matching rhizosphere colonization

The induced proteins matching rhizosphere colonization involve 31 and 15 items with assigned and unassigned biochemical features, respectively. On the first line of induced proteins are those related to protein synthesis, such as DNA transcriptional regulator [TetR family transcriptional regulator (KLL12053.1), ribosomal structure and biogenesis (WP_013874486.1/KLL10220.1; WP_047221577.1; KLL12035.1), ribonuclease PH (WP_013872713.1)], transporters and secretion systems, ATP-binding cassette (ABC) transporter (WP_013872693.1), major facilitator superfamily (MFS) transporter (KLL10396.1), SecA or type II secretory pathway (KLL10487.1) and ATPase transporters (WP_043607340.1), for diverse metabolites ranging from ions (nitrate/nitrite, iron, sulfur and potassium) to amino acids and proteins.

Amino acid-related activities supporting this active transcription and protein synthesis are also detected. A particular “loading” of tRNA with arginine, proline, cysteine, and histine affinity amino acids–tRNA ligase (WP_043602701.1; WP_013875400.1; WP_013875269.1) was noted in this study for actinorhizal RE. DNA replication, recombination and repair proteins (KLL12894.1KLL13116.1); restriction endonuclease subunit R (KLL09521.1); 5-hydroxyisourate hydrolase (KLL11137.1) may protect against cytotoxicity-generating DNA damage produced during the early step of plant defense toward microbial colonization (Baker and Orlandi, 1995; Bagnarol et al., 2007) or drastic adaptation of metabolism (Clair et al., 2012). Aldo/keto reductase (WP_050803659.1/KLL11374.1), which is an NADPH-dependent oxidoreductase, may help further detoxification of a wide range of aldehydes and ketones (Barski et al., 2008). Carbon-rich RE released by plants make the rhizosphere extremely nutrient-rich relative to the bulk soil environment (van Veen et al., 1997). Thus, the short-chain dehydrogenase/reductase (SDR) superfamily of proteins (WP_050803541.1) induced here, known as tyrosine-dependent oxidoreductases, are suggested to confer the ability to metabolize a wide range of carbon substrates that contribute to cell survival and proliferation in the nutritionally complex soil or rhizosphere environment (Jacob et al., 2008).

### Modulated proteins matching the early step receptive reply toward symbiotic recruitment

We identified, in the category of proteins suspected to be symbiotically related, 21 and 11 proteins with assigned and unassigned biochemical features, respectively. Proteins involved in the remodeling of cell surface structures are abundantly modulated. Beauchemin et al. (2012) also reported that among different RE tested, only *C. cunninghamiana* was able to alter bacterial surface properties at the fatty acid and carbohydrate levels, leading to “curling” on its compatible *F. casuarinae* strain CcI3 hyphae. In fact, microbial membrane proteins play a predominant role in bacterial auto-aggregation and host cell adhesion that is well known both for bacterial survival and the host plant colonization process (Bogino et al., 2013). Lipid metabolism proteins included cyclopropane-fatty-acyl-phospholipid synthase (KLL12366.1), which is implicated in membrane modifications (Tiricz et al., 2013), and 3-oxoacyl ACP synthase (KLL12268.1) that contributes to the biosynthesis and transfer of fatty acyl residues, which serve as acyl donors in different acyl transfers during lipid biosynthesis reactions including phospholipids (Geiger and López-Lara, 2002). Scramblase (WP_013875539.1), a calcium activation protein, is implicated in membrane depolarization through passive translocation of phospholipids between the two sides of the membrane.

Besides HisKA, and PAS sensors discussed above, a GlcNAc-PI de-N-acetylase (KLL11416.1), also named N-acetylglucosaminylphosphatidylinositol deacetylase was also induced. It catalyzes the production of peptidoglycans that contain a backbone of N-acetylglucosamine (GlcNAc) residues (Gust et al., 2012). These GlcNAc residues serve as sensing ligands to plant LysM receptor involved in early plant-microbe interactions (Rey et al., 2013). Peptidoglycan-binding lysin domain-containing protein (WP_013872588.1) may contain beside the truly secreted proteins, outer-membrane proteins, lipoproteins or proteins bound to the cell wall in a non-covalent manner (Buist et al., 2008).

In conclusion, using next-generation proteomic analysis, an overview is provided here of proteome shotgun footprinting of metabolic alterations in response to RE stimuli, which orchestrate the first steps in the recruitment of *Frankia* by compatible host plants through (i) rhizosphere colonization and then (ii) early positive perception triggering root infection and subsequent nodulation processes. In the absence of reliable methods to manipulate the genes of interest for *Frankia* strains, comparative approaches based on high-throughput gene-expression analysis after plant and bacterial stimuli provide an alternative basis for elucidating the elusive bacterial triggers in actinorhizal symbiosis.

## Author contributions

MG conceived and designed the study. AK, AG, IN, and FG performed root exudates production and *Frankia* treatments. JA and GM performed Nano liquid chromatography–MS/MS and identified the proteins. AS and IS performed bioinformatics analyses. MG, AS, and JA wrote the manuscript. All listed authors have made a substantial contribution to the work, and have approved it for publication.

### Conflict of interest statement

The authors declare that the research was conducted in the absence of any commercial or financial relationships that could be construed as a potential conflict of interest.
