# Supplementary material for: Host Plant Compatibility Shapes the Proteogenome of Frankia coriariae
Source: Front Microbiol. 2017 May 2;8:720. doi: 10.3389/fmicb.2017.00720 (PMC5411423; doi:10.3389/fmicb.2017.00720)
Supplement: Supplementary file 5 [file Table5.DOCX]

Supplementary Table S5. Proteins detected in exoproteome of strain BMG5.1 grown in BD-N medium. The datasets are a compilation of proteins generated on the basis of Dg1 and BMG5.1 genome databases. Proteins in bold were only detected in exoproteome. (-): not determined.

| **Descriptions with Accessions (Dg1/BMG5.1)** | **Pathways associated (KEGG, Brite module)** |
| --- | --- |
| methylmalonyl-CoA mutase / methylmalonyl-CoA mutase (WP_013874939.1/ KLL12803.1) | Valine, leucine a- isoleucine degradation |
| MULTISPECIES: elongation factor Tu (WP_013875114.1/ KLL12641.1) | Translation |
| acyl carrier protein / acyl carrier protein (WP_013874733.1/ KLL12911.1)  glycosyl transferase (WP_050803274.1/-)  glycosyl transferase family 2 (WP_043603010.1/-)  **methyltransferase FkbM (WP_013873088.1/-)** | Transferasesactivity |
| Crp/Fnr family transcriptional regulator (WP_013874672.1/-)  MULTISPECIES: transcriptional regulator (WP_013874517.1/-)  **transcriptional regulator / XRE family transcriptional regulator (WP_013871645.1/ KLL10923.1)**  transcriptional regulator (WP_013873150.1/-) | Transcriptionalregulation |
| elongation factor G (WP_013875115.1/-) | Transcription machinery |
| cold-shock protein (WP_013872098.1/-)  MULTISPECIES: cold-shock protein (WP_013874339.1/-) | Transcription factors |
| polynucleotide phosphorylase (WP_013874794.1/-) | Starch a- sucrose metabolism |
| **cytochrome P450 / cytochrome P450 (WP_013871958.1/ KLL10163.1)** | Signalling |
| molecular chaperone GroEL / molecular chaperone GroEL(WP_013875066.1/ KLL12682.1)  molecular chaperone GroEL / molecular chaperone GroEL(WP_013875538.1/ KLL12309.1) | RNA degradation |
| 30S ribosomal protein S12 / 30S ribosomal protein S12 (WP_013875117.1/ KLL12638.1)  30S ribosomal protein S3 / 30S ribosomal protein S3 (WP_013875106.1/ KLL12648.1)  30S ribosomal protein S4 (WP_013875087.1/ KLL12667.1)  30S ribosomal protein S7 (WP_013875116.1/-)  50S ribosomal protein L22 (WP_013875107.1/-)  50S ribosomal protein L3 /( WP_013875112.1/-)  50S ribosomal protein L4 (WP_013875111.1/-)  50S ribosomal protein L5 (WP_013875100.1/-)  50S ribosomal protein L6 / 50S ribosomal protein L6 (WP_013875097.1/ KLL12656.1)  50S ribosomal protein L7/L12 (WP_013875123.1/-)  MULTISPECIES: 30S ribosomal protein S17 (WP_043604042.1/-)  MULTISPECIES: 30S ribosomal protein S19 / 30S ribosomal protein S19 (WP_013875108.1/ KLL12647.1)  MULTISPECIES: 50S ribosomal protein L1 (WP_013875125.1/ KLL12633.1)  MULTISPECIES: 50S ribosomal protein L11 (WP_013875126.1/-) | Ribosome formation |
| extracellular ligand-binding receptor / hypothetical protein (WP_013873460.1/ WP_052914939.1) | Quorum sensing |
| adenylosuccinatelyaseadenylosuccinatelyase(WP_050803624.1/ KLL12334.1) | Purine metabolism |
| DNA-directed RNA polymerase subunit beta | Purine metabolism |
| MULTISPECIES: proteasome subunit alpha (WP_013873557.1/-)  proteasome subunit beta (WP_043603462.1/-) | Proteasome |
| bacterioferritin(WP_013874230.1/-)  **cobalamin biosynthesis protein (WP_013873628.1/-)** | Porphyrin a- chlorophyllmetabolism |
| D-alanyl-D-alanine carboxypeptidase / D-alanyl-D-alanine carboxypeptidase (WP_013872185.1/ KLL10876.1) | Peptidoglycanbiosynthesis |
| ATP synthase subunit alpha (WP_013875001.1/-)  MULTISPECIES: F0F1 ATP synthase subunit beta (WP_013874999.1/-) | Oxidative phosphorylation |
| transcription-repair coupling factor (WP_013872247.1/-) | Nucleotide excision repair |
| branched chain amino acid aminotransferase / branched-chain amino acid aminotransferase (WP_013874910.1/ KLL12820.1) | Metabolicpathways |
| ATP-dependent Clp protease ATP-binding subunit ClpX / ATP-dependent protease (WP_013872945.1/ KLL11516.1) | Heatshockproteins |
| dihydrolipoyl dehydrogenase / dihydrolipoamide dehydrogenase (WP_013874419.1/ KLL10235.1)  MULTISPECIES: co-chaperone GroES(WP_043604033.1/ KLL12681.1)  -/acetyltransferase (-/KLL11547.1) | Glycolysis / Gluconeogenesis |
| aldehyde dehydrogenase (WP_013874665.1/-)  glycine dehydrogenase (WP_013872201.1/-) | Glycine, serine a- threoninemetabolism |
| aminopeptidase N (WP_013872922.1/ KLL11531.1) | Glutathionemetabolism |
| methylthioribulose-1-phosphate dehydratase / fructose-bisphosphate aldolase (WP_013871985.1/ KLL10559.1) | Cysteine a- methioninemetabolism |
| aconitate hydratase (WP_013873515.1)  isocitratedehydrogenase, NADP-depe-ent(WP_013873302.1)  malate dehydrogenase (WP_013875039.1) | Citrate cycle (TCA cycle) |
| MULTISPECIES: type I glutamate--ammonia ligase / glutamine synthetase(WP_013874430.1/ KLL10002.1) | Arginine biosynthesis |
| arginine--tRNA ligase arginyl-tRNAsynthetase(WP_043602701.1/ KLL10060.1) | Aminoacyl-tRNAbiosynthesis |
| chemical-damaging agent resistance protein C / chemical-damaging agent resistance protein C (WP_013874969.1/ KLL11371.1) | Amino acid metabolism |
| MULTISPECIES: superoxidedismutase(WP_013873466.1/ KLL09931.1)  MULTISPECIES: superoxidedismutase, Ni (WP_013872465.1/ KLL11726.1) | Acting on superoxide as acceptor |
| ABC transporter ATP-binding protein / sulfate ABC transporter ATP-bindng protein (WP_013871647.1/ KLL10925.1)  hypothetical protein / membrane protein (WP_043607069.1/ KLL12788.1)  ABC transporter (WP_013873146.1/-)  ABC transporter substrate-bindng protein (WP_013875374.1/-) | ABC transport system |
| aminoglycoside phosphotransferase / hypothetical protein | - |
| aspartate aminotransferase (WP_013875129.1) | - |
| hypothetical protein (WP_013871882.1/-) | - |
| **hypothetical protein (WP_013874114.1/-)** | - |
| **hypothetical protein (WP_013874650.1/-)** | - |
| **hypothetical protein / hypothetical protein (WP_043603047.1/ KLL12018.1)** | - |
| **hypothetical protein (WP_043603866.1/-)** | - |
| **hypothetical protein (WP_043603974.1/-)** | - |
| **hypothetical protein (WP_050803485.1/-)** | - |
| hypothetical protein (WP_050803499.1/-) | - |
| hypothetical protein / hypothetical protein (WP_050803590.1/ WP_052914249.1) | - |
| **luciferase family oxidoreductase group 1 / FMN-linked alkanal monooxygenase (WP_013872793.1/ KLL09975.1)** | - |
| **maturase (WP_013871683.1/-)** | - |
| MULTISPECIES: chemical-damaging agent resistance protein C (WP_013872102.1/ KLL11371.1) | - |
| MULTISPECIES: Lsr2 family protein (WP_013874753.1/-) | - |
| MULTISPECIES: MaoC family dehydratase / dehydratase (WP_013872364.1/ KLL13021.1) | - |
| phosphopyruvate hydratase enolase (WP_013872252.1/ KLL13106.1) | - |
| **pilus assembly protein TadB (WP_013872600.1/-)** | - |
| putative DNA-bi-ing protein / hypothetical protein (WP_013874747.1/ KLL12904.1) | - |
| serine hydroxymethyltransferase(WP_013875009.1/-) | - |
| **SpoVT/AbrB domain-containing protein (WP_013873973.1/-)** | - |
| **-/hypothetical protein (-/WP_052914783.1)** | - |
| **-/ major capsid protein (-/ WP_047222947.1)** | - |
| **-/ PEP-utilizing protein mobile subunit (-/KLL10057.1)** | - |
| **-/prevent-host-death protein (-/KLL10298.1)** | - |
| **-/lantibiotic dehydratase (-/KLL10589.1)** | - |
| **-/NADPH-quinone reductase (-/KLL11390.1)** | - |
| **-/hypothetical protein (-/WP_047223392.1)** | - |
| **-/GGDEF domain-containing protein (-/WP_052914312.1)** | - |
| **-/hypothetical protein (-/WP_052914846.1)** | - |
| **-/CdaR family transcriptional regulator (-/WP_052914851.1)** | - |
| **-/hypothetical protein (-/KLL11626.1)** | - |
| **-/hypothetical protein (-/WP_052914985.1)** | - |
| **-/hypothetical protein (-/KLL11580.1)** | - |
| **-/glycosyl transferase (-/KLL12312.1)** | - |
